# Transcriptomic analysis of fruit stored under cold conditions using controlled atmosphere in *Prunus persica* cv. “Red Pearl”

**DOI:** 10.3389/fpls.2015.00788

**Published:** 2015-09-29

**Authors:** Dayan Sanhueza, Paula Vizoso, Iván Balic, Reinaldo Campos-Vargas, Claudio Meneses

**Affiliations:** ^1^Facultad Ciencias Biológicas, Centro de Biotecnología Vegetal, Universidad Andres BelloSantiago, Chile; ^2^FONDAP Center for Genome RegulationSantiago, Chile

**Keywords:** RNA-Seq, mealiness, nectarine, postharvest

## Abstract

Cold storage (CS) can induce a physiological disorder known as chilling injury (CI) in nectarine fruits. The main symptom is mealiness that is perceived as non-juicy fruit by consumers. Postharvest treatments such as controlled atmosphere (CA; a high CO_2_ concentration and low O_2_) have been used under cold conditions to avoid this disorder. With the objective of exploring the mechanisms involved in the CA effect on mealiness prevention, we analyzed transcriptomic changes under six conditions of “Red Pearl” nectarines by RNA-Seq. Our analysis included just harvested nectarines, juicy non-stored fruits, fruits affected for CI after CS and fruits stored in a combination of CA plus CS without CI phenotype. Nectarines stored in cold conditions combined with CA treatment resulted in less mealiness; we obtained 21.6% of juice content compared with just CS fruits (7.7%; mealy flesh). RNA-Seq data analyses were carried out to study the gene expression for different conditions assayed. During ripening, we detected that nectarines exposed to CA treatment expressed a similar number of genes compared with fruits that were not exposed to cold conditions. Firm fruits have more differentially expressed genes than soft fruits, which suggest that most important changes occur during CS. On the other hand, gene ontology analysis revealed enrichment mainly in metabolic and cellular processes. Differentially expressed genes analysis showed that low O2 concentrations combined with cold conditions slows the metabolic processes more than just the cold storage, resulting mainly in the suppression of primary metabolism and cold stress response. This is a significant step toward unraveling the molecular mechanism that explains the effectiveness of CA as a tool to prevent CI development on fruits.

## Introduction

*Prunus persica* (L.) Batsch var. nectarina is one of the world's most important temperate fruit trees due to its fruit's high nutritional value and pleasing taste (Lauxmann et al., 2012). After harvest, low-temperature storage is widely employed to overcome rapid fruit deterioration at room temperature, which thereby prolongs shelf life (Dhanapal and Crisosto, 2013), reduces enzymatic activity, and slows down respiration (Lauxmann et al., 2012). However, cold storage (CS) of nectarines leads to chilling injury (CI) that manifests itself as mealiness, flesh browning and reddening, which limits the storage period (Puig et al., 2015). The negative impacts of CS affect fruit quality (Pavez et al., 2013), particularly susceptible varieties such as late-harvest nectarine cultivars stored at low temperatures for more than 2–3 weeks (Zhou et al., 2000). CI development is faster and more intense when susceptible varieties are stored at temperatures between 2.2 and 7.6°C (Lurie and Crisosto, 2005). These symptoms largely develop after CS during ripening, and the problems are not evident until the consumer eats the fruit because symptoms affect the pulp and not the peel of the fruit (Lurie, 1992; Lurie and Crisosto, 2005; Puig et al., 2015).

Mealiness is characterized by a dry, lack of juice and mealy flesh (Lurie, 1992; Lurie and Crisosto, 2005). Traditionally, mealiness has been related to an incorrect cell wall disassembly (Brummell et al., 2004; Lurie and Crisosto, 2005). In mealy fruits, the most easily extractable cell wall pectins (water soluble) are reduced, but they have a higher molecular weight and viscosity than in juicy fruit. These high-molecular weight pectins form a gel structure that binds free water and causes the mealy phenotype (Zhou et al., 2000). Cell wall changes have been related to an imbalance in the activities of cell wall-degrading enzymes, which leads to an accumulation of de-methylesterified pectins that are not subsequently depolymerized (Obenland et al., 2003; Lauxmann et al., 2012). De-methylesterified pectins are involved in the wall in cell-to-cell adhesion, which is largely achieved by calcium cross-linking between homogalacturonan in the middle lamella (Jarvis et al., 2003). It has been suggested that mealiness is caused either by gel structures, or that the decreased intercellular adhesion in mealy fruits reduces cell rupture during biting and chewing, which inhibits the release of cellular contents (Brummell et al., 2004; Puig et al., 2015).

The incipience of CI symptoms determines the postharvest storage/shipping potential because their presence reduces consumer acceptance. Susceptibility to CI depends on genetic background, maturity, and orchard factors (Lurie and Crisosto, 2005). With the aim of avoiding CI, the industry has developed several pre and postharvest treatments, such as controlled atmosphere (CA; elevated CO_2_ and reduced O_2_ levels) (Gorny and Kader, 1996; Zhou et al., 2000; Girardi et al., 2005; Lurie and Crisosto, 2005; Lara et al., 2011), conditioning (i.e., delayed cooling; Zhou et al., 2000; Lurie and Crisosto, 2005) and intermittent warming (Girardi et al., 2005; Lurie and Crisosto, 2005). However, the effectiveness of these technologies differ in each variety and mechanisms of how these strategies alleviate CI remain unknown.

The majority of studies related to nectarine CA storage have found that reduced O_2_ and elevated CO_2_ levels confer benefits on the fruit and delay or prevent the appearance of CI symptoms. Increasing CO_2_ and decreasing O_2_ in the atmosphere around the fruit tissue has significant effects on cellular metabolism (Lurie and Crisosto, 2005). In apples, it has been reported that reduced O_2_ and/or elevated CO_2_ atmospheres reduce ethylene biosynthesis by delaying and suppressing the expression of ACC synthase on the transcriptional level and by reducing the abundance of ACC oxidase protein (Gorny and Kader, 1996). Therefore, ethylene is inhibited during storage in CA, but will recover after the fruit is exposed to air again. CA also affects the cell wall-degrading enzymes that are responsible for fruit disassembly and whose imbalance or inhibition is associated with the development of mealiness (Lurie and Crisosto, 2005). On the other hand, the respiration rate reduction in peaches has been related to CI prevention (Lurie, 1992). In this sense, the high CO_2_ content in CA storage may reduce the respiration of nectarines, which defers CI development (Lurie, 1992). However, up until now the effects and/or changes that explain the positive effect of postharvest treatments such as CA have remained unknown.

Different groups have investigated candidate genes for CI disorder in *Prunus* by analyzing transcriptomic changes under CS (González-Agüero et al., 2008; Vizoso et al., 2009; Pavez et al., 2013; Puig et al., 2015). With regards to the cell wall changes after CS in CA-treated fruit, these changes seem to be similar to the changes that occur during ripening without storage when mealiness did not develop (Zhou et al., 2000). In terms of cell wall-remodeling enzymes, an increase in mRNA polygalacturonase (PG) abundance during ripening has been reported. This increase has also been reported for cold-stored fruits in combination with CA, which results in sound flesh after ripening (Zhou et al., 2000). In CA-treated fruits, the activity of pectin esterase (PE) and exo- and endo-PG were low during CS. After ripening, the exo-PG activity in CA fruit was much higher than in fruit stored in regular air. CA resulted in the highest PG/PE ratio compared with fruit stored at low temperature in air (Zhou et al., 2000). These kinds of transcriptomic changes provide hints about the protective effect of CA.

Thanks to advances in the sequencing technology, currently it is possible to sequence the DNA in a fast, massive, and inexpensive way. RNA-Seq involves direct sequencing of cDNA using next generation sequencing. The level of transcription from a particular genomic region is estimated from the redundancy of reads (Mortazavi et al., 2008). This technology represents the latest and most potent tool for studying transcriptomes (Wang et al., 2013a). In *Prunus*, these tools have been used in peach, apricot and plum for identifying candidates genes related to PPV resistance, graft incompatibility, fruit quality, and flowering time (Martínez-Gómez et al., 2011).

In this work, we analyzed transcriptomic data obtained from “Red Pearl” nectarine fruits stored under CA. Our analysis was focused on a comparison of different conditions such as ripening and CS and the effect of CA on mealiness prevention. Because of the connection between the use of CA and the reduction in ethylene synthesis and hypoxia, we focused our search on genes related to these processes as well as changes in the cell walls. Additionally, we identified candidate genes that could explain the positive effect of CA on mealiness prevention.

## Materials and methods

### Fruit samples

**Figure 1:**
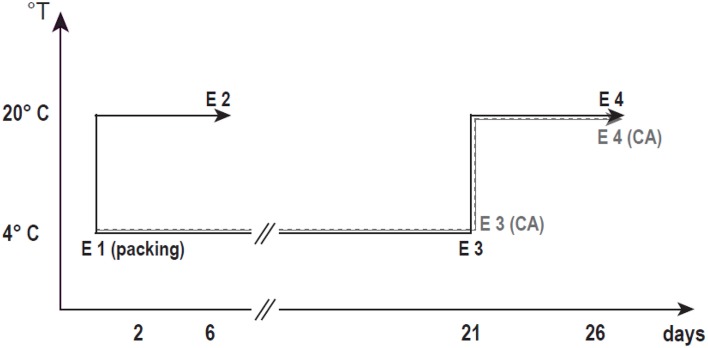
**Schematic of the evaluated postharvest conditions**. In order to understand the CA effect on CI prevention, we assayed two CS conditions (with or without CA, shown as dotted or dotted solid lines, respectively). E1 fruits came directly from the field (according to harvest parameters); E2 fruits were ripened at 20°C after harvest (less than 8.9 N firmness). E3 fruits were cold stored for 21 days at 4°C; E3CA fruits were similar to E3 fruits with the addition of CA (15% CO_2_ + 5% O_2_). E4 fruits were stored at 4°C for 21 days and then ripened at 20°C (less than 8.9 N of firmness). E4CA fruits were similar to E4 fruits with the addition of CA.

The experiments were carried out using a clingstone nectarine, “Red Pearl”. The samples were picked at the end of January from a commercial orchard located in Rengo, VI Region, Chile. The fruits were harvested at the mature stage and packed the same day. The fruit ground color was considered as the harvest index parameter (Nilo et al., 2012). After harvest, the mature fruits were transported to the laboratory and selected for uniform size and the absence of defects (E1) (Figure 1). Some of the fruit were ripened at 20°C until commercial firmness (E2), and other groups of fruits were stored at 4°C for 21 days with or without CA (15% CO_2_ + 5% O_2_) (E3CA and E3, respectively). After 21 days, the fruits were kept at 20°C until the firmness was around 8.9 N (ready to eat); this duration was considered to be the shelf life with or without CA (E4CA and E4).

We measured physiological parameters in 15 fruits at harvest, after CS and after the shelf life had elapsed. Firmness was measured on two paired sides of each fruit, which had previously been peeled to remove the epidermis, using a penetrometer fitted with an 8 mm diameter plunger. We determined the total soluble solids (TSS) content using a digital refractometer (Atago, Tokyo, Japan) (Campos-Vargas et al., 2006). Titratable acidity (TA) was determined by titrating 5 mL of juice to a pH of 8.2 with 0.1 N NaOH and expressing the result as a malic acid percentage (Girardi et al., 2005). We determined ethylene and respiration rate by enclosing individual fruit in a 750 mL jar and then sampling the air in the container. We measured ethylene by injecting 1 mL headspace sample into a gas chromatograph (GC-2014, Shimadzu, Japan) equipped with a flame ionization detector and an alumina column (Tonutti et al., 1991). We measured carbon dioxide concentrations from a headspace sample using a headspace analyzer (CheckMate 3, PBI Dansensor, Denmark) (Løkke et al., 2011). We performed a quantitative determination of mealiness using the procedure described by Crisosto and Labavitch (2002); this determination was conducted when fruits reached a softening equivalent to the eating stage (around 8.9 N). Fruits with no more than 10% juice content w/w after the quantitative measurement were considered to be mealy (Campos-Vargas et al., 2006).

### Total RNA isolation, integrity, and quantification

We extracted total RNA from mesocarp using the Hot Borate method (Gudenschwager et al., 2012). We measured the concentration and quality of the RNA using a Nanodrop (Epoch, Biotek, VT, USA) by spectrophotometric absorbance at 260 and 280 nm; the minimum accepted quality parameter was a 260/280 ratio greater than 1.8. We estimated the integrity by electrophoresis on 1.2% agarose gel under denaturing conditions.

Twelve pools of 40 μg of total RNA (12 pools = 6 conditions × 2 duplicates) were prepared and concentrations were measured using a Qubit RNA BR assay kit (Invitrogen, CA, USA) according to the manufacturer's directions. We evaluated RNA integrity using a Fragment Analyzer Automated CE System (Advanced Analytical Technologies, Inc., IA, USA) following the protocol indicated by the manufacturer. The RNA samples with RNA integrity number greater than eight were sent to sequencing facilities at Macrogen Inc. (Korea).

### RNA-seq library construction and sequencing

We isolated poly(A) mRNA using 1 μg of total RNA from each pool. The poly(A) mRNA was used to prepare a non-directional Illumina RNA-Seq library with a Tru-Seq RNA Kit according to the manufacturer's instructions (Illumina, CA, USA). We performed library quality control with a Fragment Analyzer Automated CE System using the protocol indicated by the manufacturer. We sequenced 12 libraries using HiSeq 2000 platform (Illumina) in paired-end (2 × 100 bp).

### Read processing and annotation

The reads were trimmed for base quality considering that Q ≤ 20 and reads with lengths ≤ 15 bp were removed. Paired-end reads with at least 10 bp overlapping were selected. Filtered reads were mapped against a *P. persica* reference genome sequence (Ppersica v1.0; Verde et al., 2013) using Tophat 2.0.10 (Trapnell et al., 2012). To count the mapped reads, we used the HTSeq package (http://www-huber.embl.de/users/anders/HTSeq/doc/overview.html).

Unigenes published for Ppersica v1.0 were re-annotated using BLASTX against the NCBI Nr database (Jan 2014 release), Swiss-prot (Dec 2013 release), KEGG and COG (Dec 2013 release) protein databases (cut-off *E*-value ≤ 10^−5^). InterProScan analyses were integrated using Blast2GO software (v2.7.0) (Conesa et al., 2005). We performed Gene Ontology annotation and E.C. number using Blast2GO software. The GO categorization results were expressed as three independent hierarchies for molecular functions, biological processes, and cellular components. We used AgriGO software for the statistical analyses of the gene ontology data. The KEGG pathway annotation and differential expression analyses were visualized using Degust software (http://victorian-bioinformatics-consortium.github.io/degust/).

### Principal component analysis

We carried out principal component analysis (PCA) to reveal the relationship among the treatments using Gene Expression Similarity Investigation Suite v.1.7.6 (Sturn et al., 2002). We performed PCA with the gene frequencies based on the multivariate method, and we measured the sampling adequacy by the Kaiser-Meyer-Olkin (KMO) method. We performed the validation using FactoMineR.

### Differential gene expression statistic for mRNA-seq

To identify the differentially expressed genes (DEG) between two conditions, we used the EdgeR package (Robinson et al., 2010; Anders et al., 2013). The data were normalized by the RPKM method (Mortazavi et al., 2008), and the RPKM data were used to quantify the relative gene expression. In order to determine significant differences in gene expression, we used a false discovery rate (FDR) cutoff of 0.05 as the threshold.

### Validation by qPCR analysis

Polymerase chain reactions (PCR) were carried out using the Fast EvaGreen qRT-PCR MasterMix (Applied Biotium, CA, USA) and the Mx3000P Real-Time PCR System (Stratagene, CA, USA). The experiments were performed with 3 biological replicates (performed in triplicate) that contained 25 ng cDNA, 500 nM primers, 1 × concentration Fast EvaGreen qRT-PCR MasterMix and nuclease free water with a final volume of 10 μL per reaction. To compare the data from different cDNA samples, the Ct values for all genes were normalized to the Ct value of the transcriptional elongation factor II (TEF II) (Tong et al., 2009) in each experimental condition. The results obtained for the target genes were normalized using the “Delta–delta method” (2^−ΔΔCT^) with an efficiency correction (Pfaffl, 2001).

## Results

### Physiological parameters and mealiness phenotyping

In order to determine the physiological parameters of the “Red Pearl” nectarine, fruits from all of the experimental conditions (E1, E2, E3, E3CA, E4, and E4CA) were phenotyped. Fruits stored for 21 days at low temperature (E3) were removed from this condition and then ripened (E4), which resulted in mealy fruits. During this process, a significant decrease in firmness was measured (51 and 8 N, respectively; Table 1); we also recorded a significant increase in ethylene synthesis (0.6 and 173.3 μL C_2_H_4_ kg^−1^H^−1^, respectively) and respiration rate (19 and 87.4 mL CO_2_ kg^−1^H^−1^, respectively; Table 1). The firmness was similar for all soft conditions (E2, E4, and E4CA), but significant differences in both ethylene synthesis and respiratory rate were observed, which were considerably higher in mealy fruits than the juicy nectarines. CO_2_ production was statistically the same (*p* < 0.01) in sound fruit (E2 and E4CA); ethylene was the lowest in the E2 group, intermediate in E4CA and highest in the E4 group.

**Table 1:** **Fruit maturity and physiological parameters**.

**Stage**	**Firmness (N)**	**SSC (%)**	**TA (%)**	**Juice (%)**	**Ethylene (μL/kgH)**	**Respiration rate (mL/kgH)**
E1	57.0 ± 8.3^a^	13.3 ± 1.8^a^	0.8 ± 0.1^a^		0.6 ± 0.7^c^	36.0 ± 9.6^c^
E2	12.3 ± 1.7^b^	13.8 ± 0.9^a^	0.9 ± 0.1^a^	22.4 ± 7.8^a^	47.3 ± 14.3^bc^	27.5 ± 4.2^c^
E3	51.0 ± 8.9^a^	13.4 ± 1.5^a^	0.5 ± 0.1^c^		0.6 ± 0.1^c^	19.0 ±3.8^c^
E4	8.0 ± 3.4^b^	11.9 ± 1.6^a^	0.7 ± 0.2^ab^	7.7 ± 3.7^b^	173.3 ± 107.8^a^	87.4 ± 13.0^a^
E3CA	54.0 ± 4.6^a^	13.5 ± 1.1^a^	0.6 ± 0.1^bc^		0.0 ±0.0^c^	55.7 ± 6.9^b^
E4CA	8.0 ± 1.6^b^	12.8 ± 1.1^a^	0.8 ± 0.1^a^	21.6 ± 6.5^a^	110.4 ± 24.8^ab^	28.1 ± 1.7^c^

*SSC, Soluble solids Content; TA, titratable acidity*.

a, b, c *Values (means) followed by different letters are significantly different within the same column at p ≤ 0.05*.

In order to determine mealiness, we compared the juice content in the E2, E4, and E4CA groups (Table 1). E2 fruits were juicy with an average juice content of 20.7%. The E4 fruits yielded juice content of only 7.7%, which was classified as mealy. In contrast, E4CA fruits exhibited juice content of 21.6% (Table 1 and Supplementary Material Presentation 1), which as significantly different from the E4 fruit and was similar to the E2 fruits (control). The results of measuring the juice content revealed that CA was a successful treatment for mealiness prevention in “Red Pearl” fruits.

### Sequencing, assembly, and differentially expressed genes

We sequenced 12 cDNA libraries and obtained 149.2 million paired-end reads with an average length of 100 bp (Table S1). This data set is available in the NCBI SRA database (accession number project: SRP060244, accession number sample: SRS977062). The reads were aligned by Tophat against a *P. persica* reference genome v1.0 (Verde et al., 2013), and we obtained approximately 93% uniquely mapped reads. The number of transcripts was highly reproducible and uniform between replicates for each gene (Table S1), and the Pearson correlation coefficients among biological replicates exceeded 0.97 for all comparisons.

### PCA of gene expression

**Figure 2:**
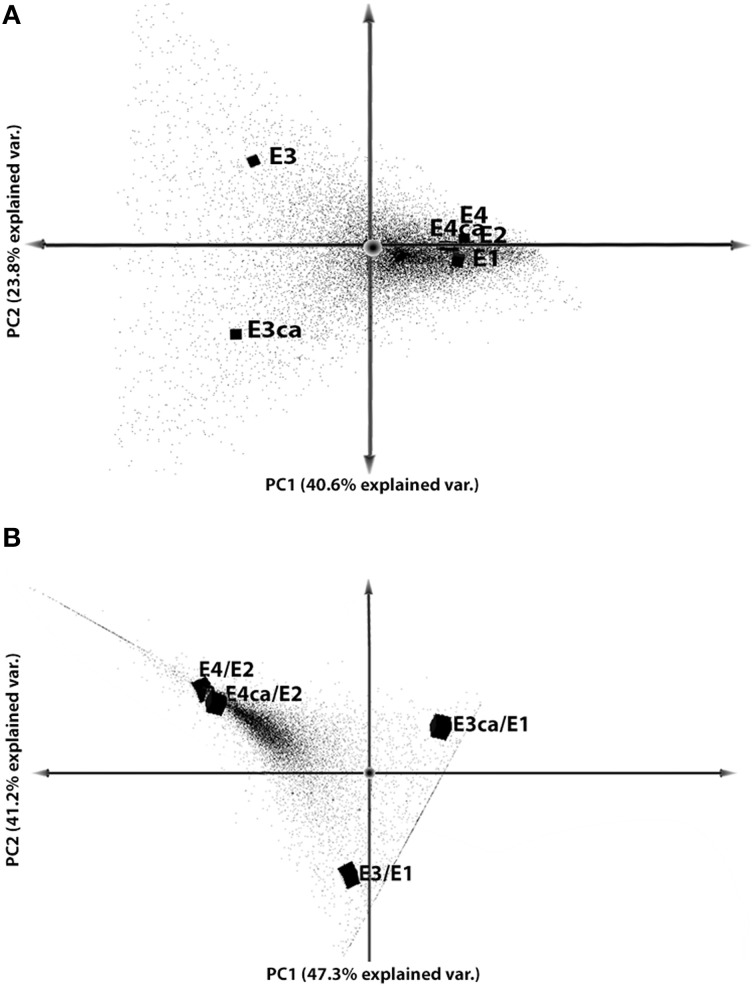
**Principal component analyses**. Dot plots of the first two components for each gene. Gene expression values were used for principal component analysis with RPKM ≥ 1, FC > 2, and FDR ≤ 0.05. **(A)** PCA of gene expression data was plotted among conditions (E1, E2, E3, E3CA, E4, and E4CA). **(B)** PCA from gene expression data between CS with or without CA (E3 and E3CA) and mature fruit (E1) and ripened with CS with or without CA (E4 and E4CA) compared with ripe fruit (E2).

To understand the gene expression variability between treatments, we performed PCA on the data set (Figure 2). We compared fruits with or without CA treatment, including ripe (E2, E4, and E4CA) and mature (E1, E3, and E3CA) fruit. This analysis explained 64.4% of the variability considering two principal components. The first component (40.6%) split CS-related genes with the other treatments, and the second component (23.8%) separated the CA effect in cold-stored fruits (Figure 2A). We validated these results with a correlation matrix obtained from gene expression analyses (Figure S1).

In a second analysis, soft cold-stored fruits with or without CA were compared with E2 fruits (E4/E2 and E4CA/E2), and firm fruit with or without CA were compared with E1 fruits (E3/E1 and E3CA/E1). The analysis explained 88.5% of the variability observed between E1 and E2 without postharvest treatments (Figure 2B). The principal aspects were concerned with the absence of differences in the expression profile between soft fruits and, on the other hand, the clear difference in gene expression between firm fruits when they were exposed to CA. The data set contained gene expression values that were exclusive for the comparisons E3CA/E1, E3/E1, E4/E2, and E4CA/E2. These DEG showed an important correlation between soft fruits with or without CA, and also but less similar behavior was observed on firm fruits. The different position on PCA observed for E3/E1 and E3CA/E1 is related with the CA effect and the dotted line that appears crossing both comparisons correspond to sequences exclusively expressed in firm fruits. On the other hand, the line observed near E4/E2 and E4CA/E2 correspond to sequences detected just in soft fruits. Here, both E4/E2 and E4CA/E2 were together on the PCA graphic showing that after CS the ripening process the gene expression was quite similar to all the fruits.

### Ripening-related genes

**Figure 3:**
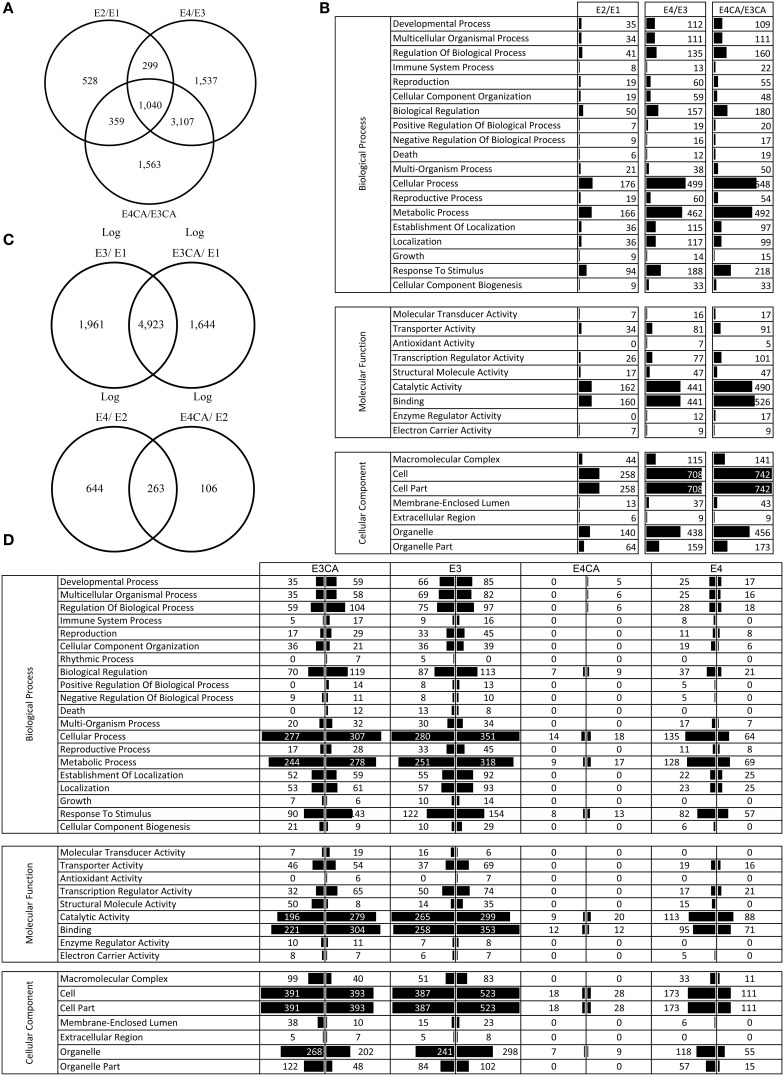
**Gene expression analyses. (A)** Venn diagram of the differential expressed genes for each comparison during ripening. Each set comprises differentially expressed genes in ripe fruit (E2/E1), ripe fruit exposed to CS (E4/E3), and ripe fruit exposed to CS and CA (E4CA/E3CA). The numbers at each intersection represent the number of genes with at least 1 RPKM. **(B)** Gene ontology classification of the exclusive expressed genes during for each ripening comparison as is shown in the Venn. **(C)** Venn diagram of genes differentially expressed in cold treatment compared with fruits exposed to CA. On the top, we display the number of genes that changed after CS with or without CA vs. E1 fruits. On the bottom, we display the number of differentially expressed genes involved in ripening after CS with or without CA (the data shown contain genes with RPKM ≥ 1 and FDR ≤ 0.05). **(D)** Gene ontology classification of differentially expressed genes with an opposite behavior to CS and CA in relation to the control conditions- E3 and E3CA against E1: 13,734 genes; E4 and E4CA in relation to E2: 2698 genes, RPKM =1, and FDR =0.05; right: up and left: down).

To identify DEG related to ripening fruit, we compared E2/E1, E4/E3, and E4CA/E3CA conditions. The DEG from the E1 to E2 groups represented a control condition for our experiment. The results revealed 2226 DEG from E1 to E2, 5983 DEG from E3 to E4 and 6069 DEG from E3CA to E4CA (Figure 3A). In the ripening process after CS, 1537 genes were exclusively detected in E4/E3, and 1563 genes were exclusively detected from E3CA to E4CA. The analysis also allowed us to identify common genes among some processes: 1040 genes were present in all ripening processes, 299 genes were common between E2/E1 and E4/E3, 359 genes were common between E2/E1 and E4CA/E3CA and 3107 genes were common between E4/E3 and E4CA/E3CA.

Using exclusively expressed genes, we conducted a GO analysis (Figure 3B). In the three comparisons (E2/E1, E4/E3, and E4CA/E3CA), metabolic process, cellular process, catalytic activity and binding were overrepresented.

### Transcriptomic changes between E3 and E3CA

To study transcriptomic changes of the CA treatment after CS, we compared E3CA and E3 conditions. We have detected 3521 DEG in which 1470 were upregulated in E3CA, and 2051 DEG exhibited the opposite behavior.

We performed the GO analysis using DEG that were exclusively up or downregulated in E3CA and E3 with E1 as the reference condition (1644 and 1961 transcripts, respectively, Figure 3C). In Figure 3D we showed functional categories with significant over-representation of some biological processes, molecular functions, and cellular components. Principal differences were observed for cellular component biogenesis, macromolecular complex, rhythmic processes, regulation of biological processes, and death. We showed 200 genes upregulated/downregulated at E3CA in Supplementary Material Table S2. In this table only genes related to ethylene biosynthesis, respiration cellular, transcription factors, and cell wall metabolism were presented in order to focus the analysis.

### CA-related genes in cold-stored ripe fruit

To analyze transcriptomic changes after ripening in cold-stored fruit, we compared E4CA and E4 fruits. We detected 328 DEG in which 133 were upregulated in E4CA and 195 exhibited the opposite behavior.

We performed the GO analysis using similar criteria than for firm fruit. We compared E4CA and E4 with E2 as reference condition (106 and 644 transcripts, respectively, Figure 3C). Figure 3D showed functional categories with significant over-representation of some biological processes, molecular functions, and cellular components. Principal differences were relative to biological processes, such as metabolic processes, response to stimulus and biological regulation. Molecular function graphs showed differences at binding and catalytic activity. Cellular component enrichment shows changes at cellular reorganization. Thirty-two genes related to ethylene biosynthesis, respiration cellular, transcription factors, and cell wall metabolism were presented in Supplementary Material Table S3.

### Gene expression validations

**Figure 4:**
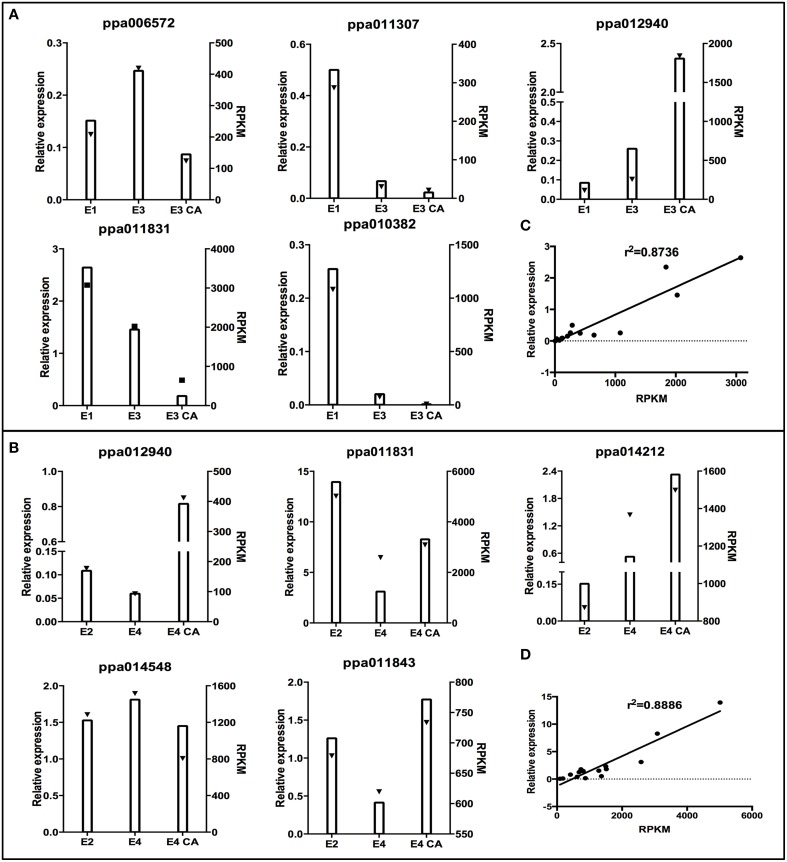
**Validation of RNA-Seq results using real-time PCR (qRT-PCR)**. To confirm the expression profile of genes in RNA-Seq results, 10 genes randomly selected from two conditions with or without treatment were analyzed. X–Y plots were generated from the ratio of transcripts levels obtained by qRT-PCR and compared with expression profile obtained by RNA-Seq (RPKM). The qRT-PCR data were obtained by analyzing the transcript level of different genes in firm and soft fruits with or without postharvest treatment, which were normalized against TEF II. The graphed qRT-PCR data represent the mean transcript level of three fruits. **(A)** Five selected genes at random with differential expression profile between E3/E3CA vs. mature fruit (E1). **(B)** Five selected genes at random with differential expression profiles between E4/E4CA vs. ripe fruit (E2). Triangles indicate RPKM values by RNA-Seq. Columns display relative expression data generated by qRT-PCR. Scatter plots show simple linear regression and the R-squared (R^2^) between relative expression obtained by qPCR (X) and RPKM values (Y) obtained by RNA-seq **(C,D)**.

To validate the expression profile obtained by RNA-Seq analysis, we performed an experimental validation by qRT-PCR on randomly selected genes from firm fruit (E1, E3, E3CA, Figure 4A) and soft fruits (E2, E4, E4CA, Figure 4B) with different levels of expression. The qRT-PCR analyses were performed using the following genes: ppa014548 (ubiquitin-protein ligase 2), ppa011843 (ATAUX2-11, IAA4), ppa011831 (plant invertase/pectin methylesterase inhibitor superfamily protein), ppa012940 (LEA14), ppa014212 (metallothionein 2B), ppa011307 (glutathione S-transferase phi 12), ppa006572 (Galactose oxidase/kelch repeat superfamily protein), and ppa010382 (expansin A8). We also conducted a simple linear regression analysis between the relative expression and RPKM; the coefficient of determination (or R-squared) was equal to 0.8736 for firm fruits (Figure 4C) and 0.8886 for soft fruits (Figure 4D). Expression profiles of the selected genes at random obtained by qPCR were consistent with the patterns of expression revealed by the RNA-Seq. The results were considered as technical validation of the DEG analysis.

## Discussion

### The effect of storage on fruit quality

The analyses of physiological parameters such as firmness, TSS, and TA revealed that none of these parameters were affected by CS or CA. TSS and TA are related strongly to fruit flavor and if no changes in these parameters means that the flavor of the fruits was not affected. With respect to ethylene production, the highest values were recorded during shelf life in the E4 and E4CA groups. In the case of CA-treated fruit, no ethylene was measured immediately after CS. However, its synthesis recovered following removal from CA to air (Lurie and Crisosto, 2005). These data suggest that CA could be affecting the fruit's metabolism (Kader, 1986). On the other hand, the highest respiration rate was recorded in mealy nectarines; CA use produces nectarines with CO_2_ levels similar to those measured in the E2 group.

With respect to mealiness development, our results were in complete agreement with previous data that described the presence of CI in late cultivars under CS (Zhou et al., 2000). We also confirmed the effectiveness of CA as a CI preventing treatment to avoid the development of mealiness in some late nectarine varieties (Lurie, 1992).

### CS and CA effects during the ripening process

The analysis of co-expressed genes during E2/E1, and E4CA/E3CA (359) displayed transcripts that are changing during the normal ripening process. Of these sequences, 246 exhibited similar gene expression profiles and 113 genes displayed opposite behavior (Figure 3A).

Otherwise, co-expressed genes during E4/E3, and E4CA/E3CA (3107) showed transcripts that are turning during the ripening process in fruit exposed to CS. Of these transcripts, 3028 exhibited the same behavior in both comparisons, and 79 genes showed the opposite gene expression profile. This group of genes that displays the opposite behavior is the most interesting because it could give us some clues about the effect of CA on the transcriptome. An example of transcripts with DEG upregulated in E4/E3 and downregulated in E4CA/E3CA were: ACC oxidase 1 (ACO1, ppa009228) related to the ethylene biosynthetic process (García et al., 2010) and xyloglucan endotransglycosylase/hydrolase 16 (XTH16, ppa020825), which participate in the carbohydrate metabolic process acting on glycosyl bonds (Supplementary Material Tables S2, S3).

### Role of CA on cold-stored fruits

As observed in the DEG analysis between the E3CA and E3 groups, more genes were downregulated than upregulated. This finding is in agreement with a previous report that found a repressive effect when fruits are stored in high CO_2_ concentrations (Balic et al., 2012). The DEG between the E3CA and E3 groups makes it possible to compare the effect of both the cold and the CA stresses. It is possible that the plant cells established a kind of hierarchy against two or more stresses and that the response was focused on the strongest of these stresses. Thus, we suggest that the low O_2_ concentration has a larger impact than the CS treatment. For this reason, we conducted a cold stress vs. CA stress comparison. Among the upregulated sequences, we found AOC4 (Supplementary Material Table S2), an enzyme that catalyzes an essential step in jasmonic acid (JA) biosynthesis (Ziegler et al., 2000). AOC4 exhibited a reduction in gene expression during senescence (He et al., 2002). AOC4 is a good example of a possible anti-senescence effect of CA on nectarines, specifically by stimulating the synthesis of JA. In peaches, reports have described that the application of methyl jasmonate vapor alleviates mealiness (Jin et al., 2009). Within the idea of transcriptional regulation, we found RDR1 to be related to the positive regulation of post-transcriptional gene silencing in response to a salicylic acid (SA) stimulus (Wang et al., 2014b). A positive effect of SA application with a concomitant reduction of CI in peaches has been described (Wang et al., 2006). We observed SAG20/101 senescence-related genes in our studies were upregulated in E3CA (Supplementary Material Table S2). SAG20 is a senescence-related sequence induced during chronic ozone (O_3_) exposure, darkness, dehydration, and treatment with ethylene or ABA. This fact suggests that all of these conditions may induce similar signaling molecules. Senescence is a highly regulated degradative process involving photosynthetic decline, protein degradation, lipid peroxidation, and chlorophyll degradation. Plant hormones are involved in regulating this process, with cytokinins delaying senescence and ethylene modulating the timing (Miller et al., 1999).

Some transcription factors involved in regulation of senescence are members of WRKY family. We found a WRKY 4, which it has been proposed as a potential key regulator in the cross talk between SA and JA pathways and besides it was related to energy production (Higashi et al., 2008). In this case, the upregulation of this gene could be related to the energy necessary to keep some cellular processes running, particularly those that are responding to the stress condition due to the high CO2 concentration. Another member is WRKY33 that is known as regulator for diverse abiotic stresses such as high salinity, osmotic stress, cold, and heat shock (Dubois et al., 2013; Wang et al., 2013b). An increase of stress-related genes when CA is used instead of regular air can be highly relevant to the cell for its ability to deal CS and hypoxia (Supplementary Material Table S2).

As expected in E3CA, there was an increase in the abundance of transcripts related to hypoxia processes. We detected DEG transcript for ADH1 and SUS3, a hypoxia-responsive proteins involved in cellular respiration, redox, the regulation of H_2_O_2_ (Yang, 2014) and glycolysis/gluconeogenesis, among other processes involved in hypoxic stress tolerance (Baroja-Fernández et al., 2012; Wang et al., 2014a).

With respect to cell wall modification, we found a member of MYB transcription factor family, which it was co-expressed directly with genes belonging to xyloglucan endotransglucosydase/hydrolase family (Supplementary Material Table S2). Another co-expressed genes as pectin lyase related to cell wall modification were found to be upregulated in E3CA. On the other hand, presence of several genes related as invertase/pectin methylesterase inhibitor (PMEI) family suggests that these proteins play a potentially important role in cell wall modifications. The de-methyl esterification effect produces free carboxyl groups modifying the pH and charges in the cell wall, which allows for the aggregation of polyuronides into a calcium-linked gel structure that increases the wall firmness and finally, it prevents the expression of the mealiness in the fruits. The specificity of PMEI for plant pectin methylesterase (PME) prompts a physiological role in the modulation of endogenous PME activity (Lionetti et al., 2007). The reduction of methyl groups in mealy fruits has been specifically described for peaches and nectarines (Lurie and Crisosto, 2005). The significant accumulation of PMEI transcripts suggests a low PME activity. This hypothesis agrees with other data obtained in our group that exhibited the presence of methyl groups in juicy fruit both in E2 and as a consequence of the use of CA; methyl groups were missing in mealy flesh (data not shown). Our results show a different transcript accumulation between treated/untreated conditions (Figures 4A,B).

As consequence of CA treatment, several transcripts related to ethylene were downregulated. This repression impacted processes such as transcription, response to stress, and cold and signal transduction. As an example of the compensatory process due to the lack of ethylene, ETR2, and ERS1 were repressed. On the other hand, ERS1 exhibits a negative regulation of ethylene-mediated pathways (Liu and Wen, 2012; Qiu et al., 2012). The repression of 1-aminocyclopropane-1-carboxylate oxidase that was involved in fruit ripening is a clear example that CA is leaving the fruit on a “stand by” state. The decrease in abundance of fructose-bisphosphate aldolase is consistent with this idea. This protein is related to the pentose-phosphate shunt, photorespiration, the response to misfolded proteins, temperature stimuli (Lu et al., 2012), and water transport. It participates in the biosynthesis of secondary metabolites, glycolysis/gluconeogenesis, the TCA cycle, carbon fixation in photosynthetic organisms, and pyruvate metabolism. With the downregulation of this sequence, potentially all of these processes may be negatively affected.

Among the downregulated genes, we found transcripts that are, for example, related to cytokinin and auxin response or signaling. It is known that both hormones are involved in diverse fruit-development processes. In this group, we also found cold response-related sequences. This situation suggests that maybe the cells classify hierarchically into different types of stresses. All of the fruits were stored in the dark at 4°C, but just some of them were exposed to CA. Perhaps the cell adopts the option of facing the condition that could potentially be the most harmful (in this case, the high CO_2_ concentration).

### CA effect in cold-stored ripe fruit

Even when conditions are phenotypically completely different—one mealy and the other juicy at the transcriptomic level they seem to be quite similar. The low level of DEG in ripe fruit may suggest that most of the changes are occurring during CS and that the observed phenotype is a consequence of changes that occur before ripening.

With respect to ethylene synthesis, AOC4 (ppa008791) was repressed in E3CA and it showed an increase during ripening in the E4CA (Supplementary Material Table S3). An increase in transcript abundance from E3CA to E4CA was also recorded for pectin lyase, a protein that participates in fruit softening. Cell wall related genes involved in xyloglucan modification and biosynthesis were upregulated. These changes suggest that hemicelluloses, instead of only pectins, are also relevant in the normal ripening process.

Other transcripts that increased in E4CA included FAD-binding Berberine (ppa025579), which is related to the redox process. These genes that are co-expressed with this sequence include WRKY 23, expansin A4, plant invertase/pectin methyltransferase inhibitor superfamily and pectin lyase-like superfamily protein, all of which are expressed during leaf senescence (Supplementary Material Table S3).

The repression in E4CA of a senescence-associated sequence such as SAG29 may be involved in a better quality fruit (Supplementary Material Table S3). This protein is expressed primarily in senescing plant tissues and is induced by osmotic stresses via an abscisic acid-dependent pathway. A study using a SAG29-deficient mutant revealed enhanced cell viability under high-salinity conditions. This evidence proposes that the SAG29 protein may serve as a molecular link that integrates environmental stress responses into senescing processes (Seo et al., 2011).

## Conclusions

PCA analysis proposes that most relevant changes related to CI development occur during CS when the fruits are still firm.

The DEG analysis revealed more downregulated genes in the E3CA group than the E3 group. This finding agrees with the idea that fruit's systems slow down when they are exposed to CA. In the E3CA group, we observed an important downregulation of ethylene-related transcripts, especially those that exhibited a negative regulation. This situation could be the effect of the low O_2_ concentration and high CO_2_ concentration in which the fruits were trying to compensate for no or little ethylene synthesis.

The ripening process analysis that produced juicy fruits revealed that just a few transcripts differed between the E4CA and E4 conditions. This fact suggests that the ripening process after CS plus CA is not significantly different from the softening process that occurs in mealy nectarines. The analyzed data revealed that the upregulated transcripts in the E4CA group were mainly focused on hemicellulose modification such as XTH and expansins. However, changes in genes related to senescence and reactive oxygen species were also detected. This situation may be indicative of the presence of a process known as burst, which has been previously described in fruits stored in CA conditions (Larrigaudiere et al., 2001).

The data presented in this work seem to indicate that in the presence of two types of stresses —namely CA and cold—cells construct a kind of hierarchy in order to respond mainly to the most important stress condition. In this case, CA seems to be stronger stress than cold. In general terms, the effect of elevated CO_2_ and/or reduced O_2_ on reducing the respiration rate has been assumed to be the basic reason for the beneficial effects of CA (Kader et al., 1989). Nevertheless, our results revealed changes that are affecting the primary metabolism (e.g., glycolysis and the TCA cycle). Therefore, our findings show that others metabolic processes are also relevant in the CA effect.

As a potential application of these data, transcriptomic changes have been useful to develop biomarkers that assess disorder susceptibility (Gapper et al., 2013). Special clues for this idea could be found in genes that exhibited an opposite profile as a consequence of CA used when fruits were cold-stored, and also in transcripts that were expressed only under CA conditions.

## Author contributions

DS: experimental design, data phenotypic acquisition and analysis, RNA extraction and gene expression analysis, bioinformatics, and statistical data analysis and drafted the manuscript. PV: experimental design, sequence alignment, bioinformatics, and statistical data analysis. IB: collaborated on the data analysis. RC: experimental design and made intellectual contributions to the manuscript. CM: experimental design, data analysis, drafted the manuscript. All of the authors read and revised the manuscript critically and approved the final version.

### Conflict of interest statement

The authors declare that the research was conducted in the absence of any commercial or financial relationships that could be construed as a potential conflict of interest.
