# Supplementary material for: Transcriptomic analysis of fruit stored under cold conditions using controlled atmosphere in Prunus persica cv. “Red Pearl”
Source: Front Plant Sci. 2015 Sep 29;6:788. doi: 10.3389/fpls.2015.00788 (PMC4586424; doi:10.3389/fpls.2015.00788)
Supplement: Supplementary file 1 [file Table1.DOCX]

**Table S1 Summary of sequencing metrics**

| Stage | Paired-end | Number of reads (M) | Mapped reads (%) | Pair alignment (%) |
| --- | --- | --- | --- | --- |
| E1 | R1 | 11.2 | 89.6 | 83.2 |
|  | R2 | 10.4 | 92.0 | 80.7 |
| E2 | R1 | 9.9 | 92.1 | 80.7 |
|  | R2 | 12.6 | 92.4 | 81.1 |
| E3 | R1 | 15.4 | 90.7 | 85.7 |
|  | R2 | 12.8 | 93.0 | 84.2 |
| E4 | R1 | 12.3 | 93.0 | 84.2 |
|  | R2 | 15.7 | 93.8 | 83.9 |
| E3CA | R1 | 11.1 | 97.3 | 76.6 |
|  | R2 | 11.8 | 92.6 | 83.1 |
| E4CA | R1 | 13.8 | 93.7 | 85.4 |
|  | R2 | 12.2 | 92.8 | 83.6 |
