# Supplementary material for: Transcriptomic analysis of fruit stored under cold conditions using controlled atmosphere in Prunus persica cv. “Red Pearl”
Source: Front Plant Sci. 2015 Sep 29;6:788. doi: 10.3389/fpls.2015.00788 (PMC4586424; doi:10.3389/fpls.2015.00788)
Supplement: Supplementary file 4 [file Presentation1.PDF]

Experimental design

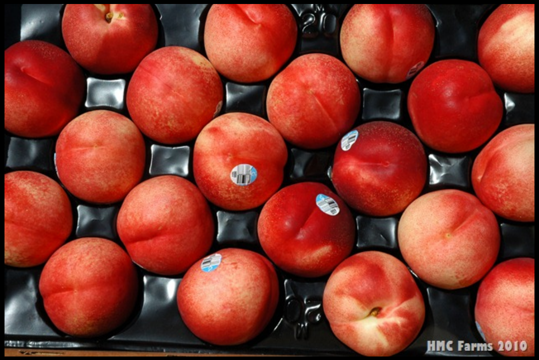

Harvest; physiologically mature; firm fruit

20 ºC; less 2 lbs

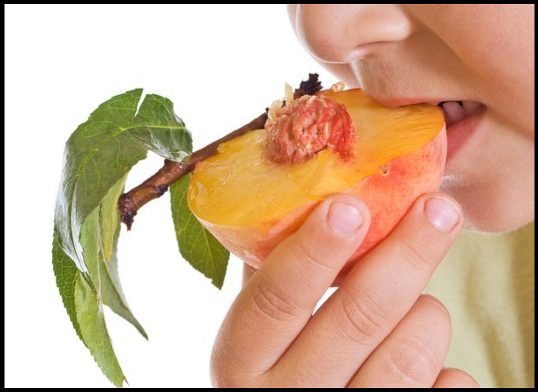

Ripe; **JUICY FRUIT**; soft fruit

21 days at 4 ºC

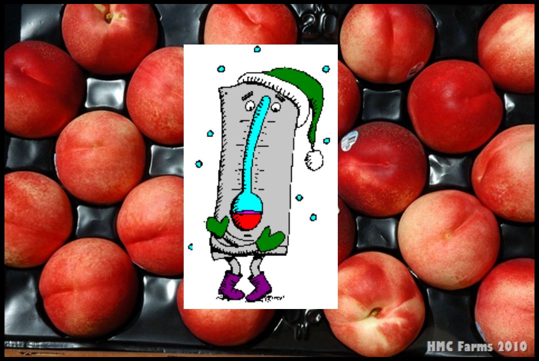

20 ºC; less 2 lbs

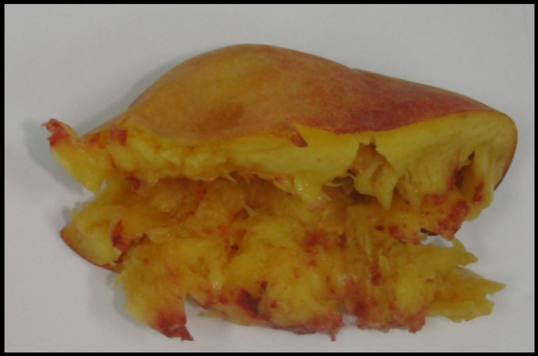

Ripe; **MEALY FRUIT**; soft fruit

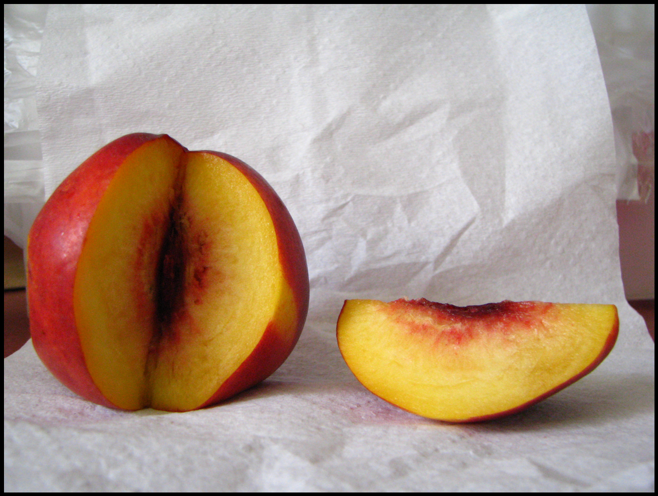

E4,
mealy

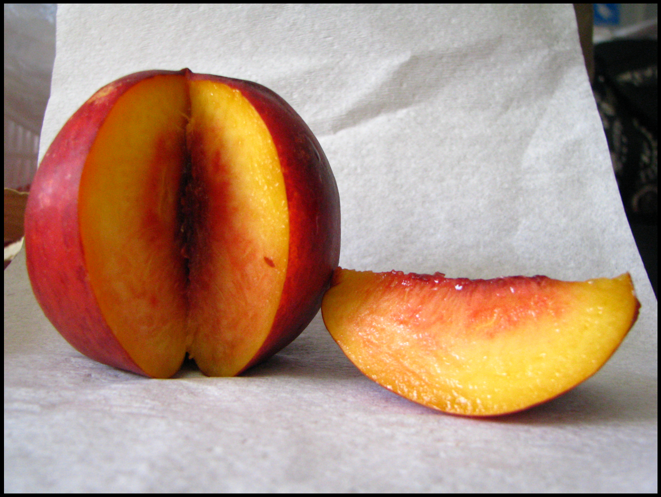

E4CA,
juicy
