# Supplementary material for: Transcriptomic analysis of fruit stored under cold conditions using controlled atmosphere in Prunus persica cv. “Red Pearl”
Source: Front Plant Sci. 2015 Sep 29;6:788. doi: 10.3389/fpls.2015.00788 (PMC4586424; doi:10.3389/fpls.2015.00788)
Supplement: Supplementary file 5 [file Image1.PDF]

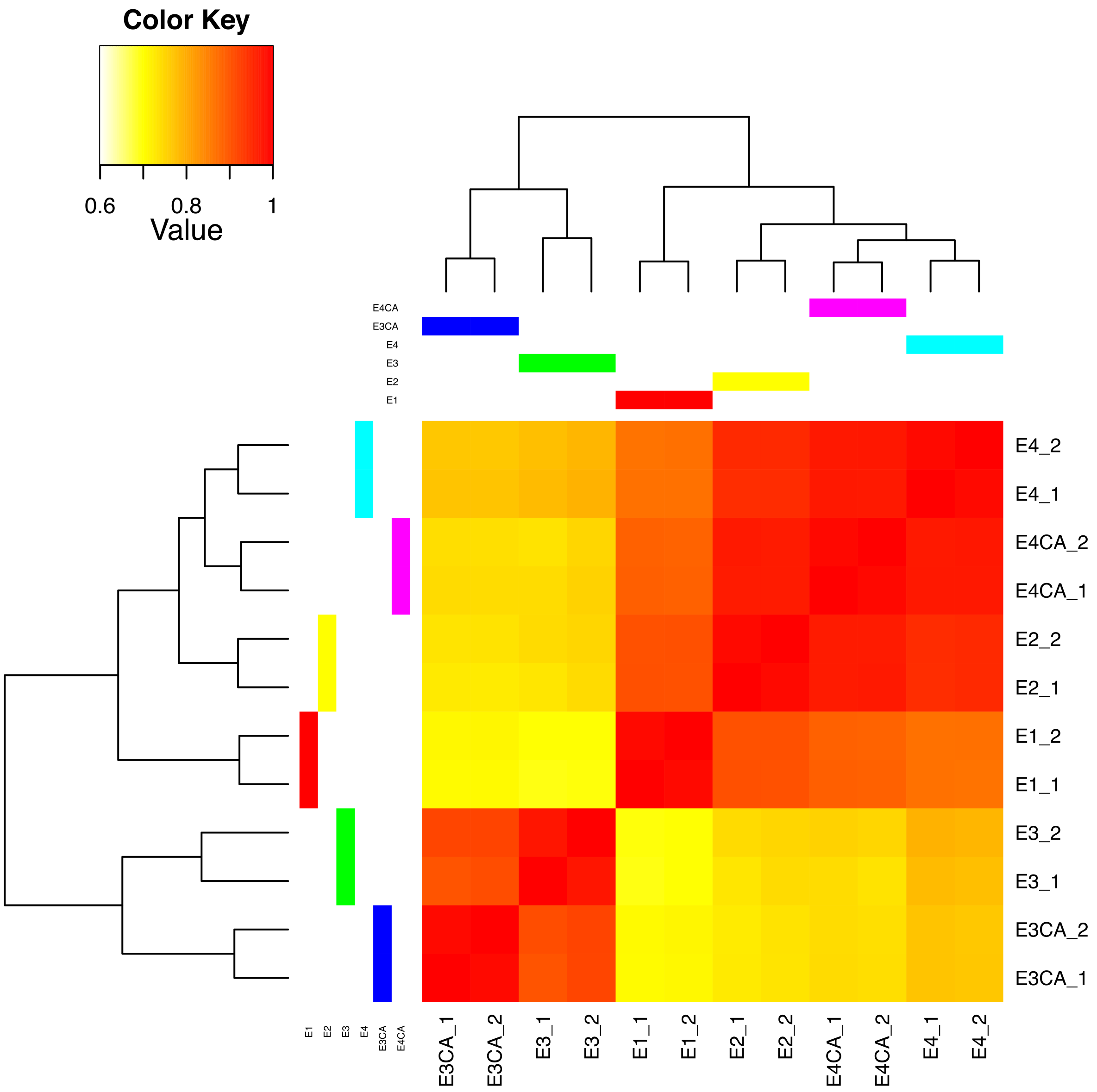

Figure S1 Correlation matrix. To validate PCA analysis a hieralchical clustering analyses (HCA) was constructed based on standardized input data and pearson distance. Gradient color (from yellow to red) shown correlation values (0.7 to 1.0).
